# Effects of polyamines and indole on the expression of ribosome hibernation factors in Escherichia coli at the translational level

**DOI:** 10.18699/vjgb-24-04

**Published:** 2024-02

**Authors:** E.A. Khaova, A.G. Tkachenko

**Affiliations:** Institute of Ecology and Genetics of Microorganisms of the Ural Branch of the Russian Academy of Sciences, Perm Federal Research Center of the Ural Branch of the Russian Academy of Sciences, Perm, Russia; Institute of Ecology and Genetics of Microorganisms of the Ural Branch of the Russian Academy of Sciences, Perm Federal Perm Federal Research Center of the Ural Branch of the Russian Academy of Sciences, Perm, Russia

**Keywords:** polyamines, polyamine modulon, indole, ribosome hibernation factors, reporter gene fusions, gene expression, adaptation to stress, полиамины, полиаминовый модулон, индол, факторы гибернации рибосом, репортерные генные слияния, генная экспрессия, адаптация к стрессу

## Abstract

Polyamines and indole are small regulatory molecules that are involved in the adaptation to stress in bacteria, including the regulation of gene expression. Genes, the translation of which is under the regulatory effects of polyamines, form the polyamine modulon. Previously, we showed that polyamines upregulated the transcription of genes encoding the ribosome hibernation factors RMF, RaiA, SRA, EttA and RsfS in Escherichia coli. At the same time, indole affected the expression at the transcriptional level of only the raiA and rmf genes. Ribosome hibernation factors reversibly inhibit translation under stress conditions, including exposure to antibiotics, to avoid resource waste and to conserve ribosomes for a quick restoration of their functions when favorable conditions occur. In this work, we have studied the influence of indole on the expression of the raiA and rmf genes at the translational level and regulatory effects of the polyamines putrescine, cadaverine and spermidine on the translation of the rmf, raiA, sra, ettA and rsfS genes. We have analyzed the mRNA primary structures of the studied genes and the predicted mRNA secondary structures obtained by using the RNAfold program for the availability of polyamine modulon features. We have found that all of the studied genes contain specific features typical of the polyamine modulon. Furthermore, to investigate the influence of polyamines and indole on the translation of the studied genes, we have constructed the translational reporter lacZ-fusions by using the pRS552/λRS45 system. According to the results obtained, polyamines upregulated the expression of the rmf, raiA and sra genes, the highest expression of which was observed at the stationary phase, but did not affect the translation of the ettA and rsfS genes, the highest expression of which took place during the exponential phase. The stimulatory effects were polyamine-specific and observed at the stationary phase, when bacteria are under multiple stresses. In addition, the data obtained demonstrated that indole significantly inhibited translation of the raiA and rmf genes, despite the stimulatory effect on their transcrip- tion. This can suggest the activity of a posttranscriptional regulatory mechanism of indole on gene expression.

## Abstract (ru)

Полиамины и индол – регуляторные молекулы, которые участвуют в адаптации к стрессу у бактерий, включая регуляцию генной экспрессии. Гены, трансляция которых находится под регуляторным влиянием полиаминов, составляют полиаминовый модулон. Ранее нами показано, что полиамины стимулируют транскрипцию генов, кодирующих факторы гибернации рибосом RMF, RaiA, SRA, EttA, RsfS у Escherichia coli, а эффект индола ограничивался лишь двумя из них – raiA и rmf. Факторы гибернации рибосом обратимо ингибируют трансляцию в условиях стресса с целью экономии клеточных ресурсов, играя ключевую роль в выживании бактерий, в том числе при воздействии антибиотиков. Данная работа посвящена изучению влияния индола на экспрессию генов raiA и rmf на трансляционном уровне, а также регуляторных эффектов полиаминов путресцина, кадаверина и спермидина на трансляцию генов rmf, raiA, sra, ettA, rsfS. Проанализировав первичные структуры мРНК, а также полученные с помощью программы RNAfold модели вторичных структур мРНК, мы установили, что все исследуемые гены имеют специфические признаки полиаминового модулона. Для изучения влияния полиаминов и индола на трансляцию исследуемых генов были сконструированы трансляционные репортерные lacZ-слияния с использованием pRS552/λRS45 системы. Согласно полученным результатам, полиамины стимулируют экспрессию «стационарно-фазных» генов rmf, raiA, sra, но не оказывают влияния на трансляцию генов ettA и rsfS, наибольшая экспрессия которых наблюдается в экспоненциальной фазе роста. Стимулирующий эффект специфичен для различных полиаминов и во всех случаях наблюдается в стационарной фазе, когда клетки подвержены множественному стрессорному воздействию. Кроме того, полученные данные демонстрируют значительный ингибирующий эффект индола на экспрессию raiA и rmf на уровне трансляции, несмотря на его стимулирующее действие на транскрипцию данных генов, что может являться признаком функционирования посттранскрипционного механизма их регуляции

## Introduction

Being the normal metabolites of bacteria, polyamines and indole are involved in a variety of cellular processes, including adaptation to stress, antibiotic resistance, biofilm formation, quorum sensing and persistence (Rhee et al., 2007; Shah, Swiatlo, 2008; Tkachenko et al., 2012, 2014; Gaimster et al., 2014; Lee et al., 2015; Miller-Fleming et al., 2015; Michael, 2018; Kim et al., 2020; Zarkan et al., 2020; Lang et al., 2021). Biogenic polyamines are aliphatic polycations synthesized from amino acids and present in almost all biological materials. Bacteria are able to produce predominantly putrescine, cadaverine and spermidine (Tabor C.W., Tabor H., 1985; Michael, 2016). In turn, indole is a heterocyclic aromatic compound, which is produced from tryptophan by many bacterial species and is involved in interspecies and interkingdom signaling (Zarkan et al., 2020).

One of the ways to realize the regulatory effects of these metabolites is the modulation of gene expression (Igarashi, Kashiwagi, 2006, 2018; Kusano et al., 2008; Shah, Swiatlo, 2008; Miller-Fleming et al., 2015; Zarkan et al., 2020; Lang et al., 2021). Intracellular polyamines are mainly presented by complexes with RNA, including mRNA. Genes, the expression of which is upregulated by polyamines at the translational level, form the polyamine modulon (Igarashi, Kashiwagi, 2006, 2018). At the same time, there are data indicating that the regulatory activity of both polyamines and indole can be displayed on different levels of gene expression (Miller-Fleming et al., 2015; Lang et al., 2021; Khaova et al., 2022). Signaling molecules are able to form the regulatory networks that can form responses to various stresses (Tkachenko, 2012). Recently, data were produced about the mutual influence of polyamines and indole on Escherichia coli metabolism. In particular, exogenous indole was able to increase the intracellular content of putrescine and spermidine, whereas the addition of spermine, which is the predominant product of eukaryotes, was capable of increasing the indole content in the cultural medium (Nesterova et al., 2021). The functioning of regulatory networks is aimed at optimizing responses of bacterial cells to changes in environmental conditions (Tkachenko, 2012). Polyamines and indole are known to have many different effects on cellular processes, but their molecular targets and mechanisms of action are still not fully understood (Rhee et al., 2007; Kusano et al., 2008; Shah, Swiatlo, 2008; Lee et al., 2015; Miller-Fleming et al., 2015; Michael, 2018; Zarkan et al., 2020).

Previously, we studied the influence of polyamines and indole on transcription of the rmf, raiA, sra, ettA, rsfS genes, encoding ribosome hibernation factors, in E. coli (Khaova et al., 2022). These factors are able to reversibly inhibit ribosomes under the conditions of nutrient depletion and other stresses in order to save cell resources and conserve ribosomes for the following rapid restoration of their functioning as soon as normal growth conditions are restored. The functioning of ribosome hibernation factors can lead to the formation of a dormant state in a bacterial cell. The dormant state is a metabolically inactive state characterized by growth arrest (Prossliner et al., 2018; Trösch, Willmund, 2019; Usachev et al., 2020).

The formation of persistence is associated with dormancy. Persisters are rare variants of regular cells that have multidrug tolerance and are one of the reasons for the recalcitrance of chronic infectious diseases (Lewis, 2010; Zhang, 2014; Balaban et al., 2019). In addition, persisters are capable of mutating and surviving during exposure to high concentrations of antibiotics and, therefore, can be considered as a “reservoir” for the emergence of resistant mutants (Zhang, 2014; Tkachenko, 2018). Although the molecular mechanisms of persistence are still poorly understood, recently a model for the formation of persisters as a result of ribosome hibernation factors’ activity has been suggested (Song, Wood, 2020). There are also data on the involvement of polyamines and indole in persistence (Tkachenko et al., 2014, 2017; Zarkan et al., 2020; Lang et al., 2021). It can be assumed that these signaling molecules are involved in the formation of persistence through modulation of the expression of genes encoding ribosome hibernation factors. This is due to an ability of polyamines to stimulate the transcription of the rmf, raiA, sra, ettA, rsfS genes encoding ribosome hibernation factors, whereas indole is able to increase the transcription of two of them, raiA and rmf (Khaova et al., 2022).

These factors have different mechanisms of action. RMF (al-ternative names – Res, RimF) together with the HPF factor (alternative name – YhbH) can form inactive 100S dimers of ribosomes. RaiA (alternative names – YfiA, pY, Urf1) is able to block the active centers of 70S ribosomes, whereas SRA (alternative names – RpsV, Protein D) inhibits the translation by interacting with the 30S subunit, and EttA (alternative name – YjjK) inactivates ribosomes in response to low intracellular levels of ATP. Finally, RsfS (alternative names – YbeB, RsfA) prevents the interaction of 50S and 30S subunits (Prossliner et al., 2018).

The aim of this work is to study the effects of indole on the expression of the raiA and rmf genes at the translational level, as well as the regulatory effects of the polyamines putrescine, cadaverine and spermidine on the translation of the rmf, raiA, sra, ettA and rsfS genes.

## Materials and methods

Strains and growth conditions. E. coli strains used in this work are listed in Table 1. The cells of strains were grown in LB broth (Sigma) or defined medium M9 (+0.4 % glucose) in thermoshaker GFL-1092 (GFL) at 37 °C and 120 rpm. Media were supplemented with kanamycin 25 μg/ml (AppliChem) and/or ampicillin 50 μg/ml (AppliChem) when required. LB broth was used for strain constructions and routine cell growth.

**Table 1.**
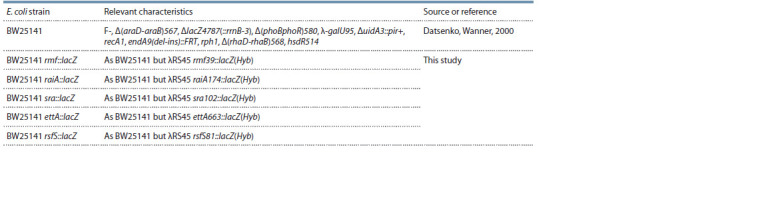
E. coli strains used in this work

For experiments studying the gene expression, cells of strains harboring lacZ-fusions were grown in defined medium M9 (+0.4 % glucose). Putrescine, cadaverine, and spermidine hydrochlorides (Sigma) were added to the medium for 2 h of cultivation at the concentrations indicated in the figures. Tryptophan (AppliChem) at 2 mM was added as previously described (Khaova et al., 2022).

Construction of the translational lacZ-fusions.

BW252141 strains with the chromosomal lacZ-fusions were obtained by using the pRS552/λRS45 system (Simons et al., 1987). Primers used in this work are listed in Table 2.

**Table 2.**
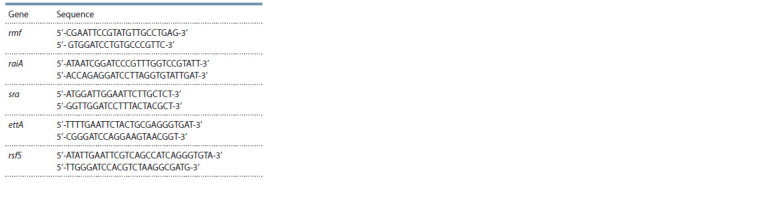
Primers used for construction of the translational lacZ-fusions

The in-between and resulting genetic constructs were verified by PCR and sequenced. Sequencing was performed by Evrogen (Moscow, Russia). All of the enzymes used were purchased from Thermo Fisher Scientific

β-galactosidase assay. Gene expression was detected by the β-galactosidase activity by Miller’s method (Miller, 1972).

mRNA secondary structure prediction. mRNA secondary structures were predicted using the RNAfold program (Lorenz et al., 2011).

Statistical analysis. Statistica for Windows 5.0 (StatSoft, Inc., 1995) software was used in the processing of experimental data, presented as mean values of 3–5 independent experiments ± standard deviation (Mean ± SD).

## Results

Polyamines are known to be able to regulate the gene expression. Genes, the expression of which is upregulated by polyamines at the translational level, are structured into the polyamine modulon. There are several mechanisms by which polyamines are able to modulate gene expression at the translational level, and, accordingly, specific features in the mRNA structure of such genes. Currently, there are data that mostly indicate the involvement of polyamines in the regulation of gene expression at the level of translational initiation. Firstly, these metabolites contribute to the initiation of the translation of genes, the mRNA structure of which contains the minor (ineffective) start codon. Secondly, polyamines stimulate the initiation of translation of genes, the mRNA structure of which has an unusually long distance between the Shine–Dalgarno sequence and the start codon. By introducing a bend in the mRNA at this region, polyamines are able to reduce this distance (Igarashi, Kashiwagi, 2006, 2018). Thirdly, polyamines are able to relax secondary structures such as “bulged-out” regions, which, being located between the Shine–Dalgarno sequence and the start codon, prevent the initiation of translation (Lightfoot, Hall, 2014).

**Fig. 1.**
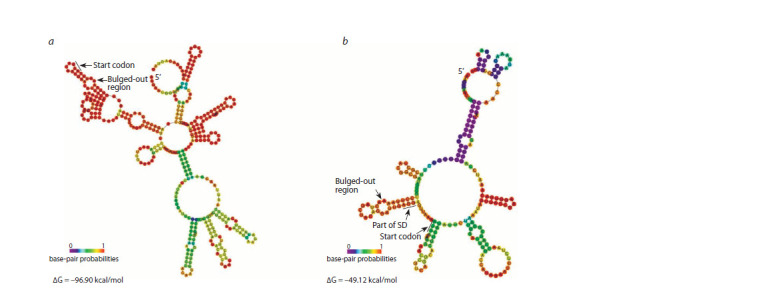
Models of mRNA secondary structures of the raiA (a) and sra (b) genes obtained using the RNAfold program (Lorenz et al., 2011). SD – Shine–Dalgarno sequence.

We have analyzed the mRNA structures of the rmf, raiA, sra, ettA, rsfS genes for the presence of the specific features of polyamine modulon genes using the GenBank and EcoCyc databases (Keseler et al., 2021) and published data, as well as the obtained models of mRNA secondary structures using the RNAfold program (Lorenz et al., 2011). According to published data, the rmf mRNA is characterized by an unusually long distance between the Shine–Dalgarno sequence and the start codon and the presence of a bulged-out structure in this region (Sakamoto et al., 2020). However, there is no information in the literature on the effect of polyamines on the expression of the remaining genes. According to the mRNA sequence of the genes, ettA mRNA and rsfS mRNA contain minor start codons GUG and UUG, respectively. The obtained models of mRNA secondary structures showed that for two genes, raiA and sra, the bulged-out structure can occur in the region of interest with a high probability (Fig. 1). The model of the raiA mRNA secondary structure demonstrates the presence of a bulged-out structure at a distance of 3 nucleotides from the start codon. According to the model obtained for the sra mRNA secondary structure, the bulged-out region comprises a part of the Shine–Dalgarno sequence. Thus, the studied genes have properties specific for polyamine modulon.

**Fig. 2.**
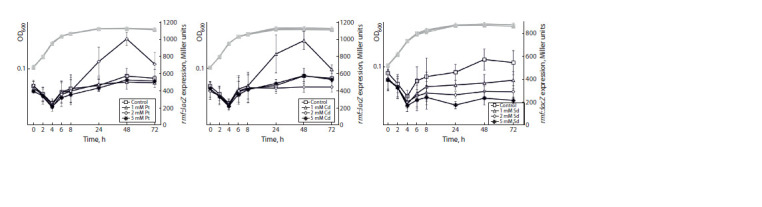
The influence of polyamine additions – putrescine (Pt), cadaverine (Cd) and spermidine (Sd) – at different concentrations on the expression of the rmf gene at the translational level. Here and elsewhere: gray curves – optical density, black curves – gene expression. OD600 – optical density at 600 nm.

Using obtained strains harboring lacZ-fusions, we studied the effects of polyamine additions at different concentrations – putrescine, cadaverine, and spermidine – on the expression of the rmf, raiA, sra, ettA, and rsfS genes at the translational level. The obtained results demonstrate that rmf gene expression is significantly stimulated by the addition of putrescine at 2 mM and cadaverine at 1 mM (Fig. 2). The maximal stimulatory effect is observed at 48 h of cultivation, when the bacterial cells are at the stationary phase. In contrast, spermidine inhibits rmf expression in proportion to the concentration of the supplement. Native rmf gene expression without additions remains at a consistently high level during the stationary phase.

**Fig. 3.**
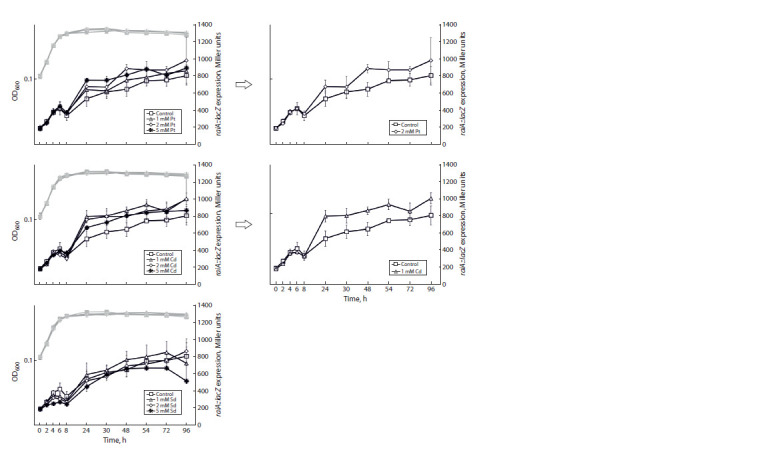
The influence of polyamine additions – putrescine (Pt), cadaverine (Cd) and spermidine (Sd) – at different concentrations on the expression of the raiA gene at the translational level. OD600 – optical density at 600 nm.

According to the results obtained, the expression of the raiA gene is at a low level during exponential growth and increases at the stationary phase (Fig. 3). Supplements of putrescine at 2 mM and cadaverine at 1 mM insignificantly increase the expression of raiA in the stationary phase, whereas spermidine has no effect.

**Fig. 4.**
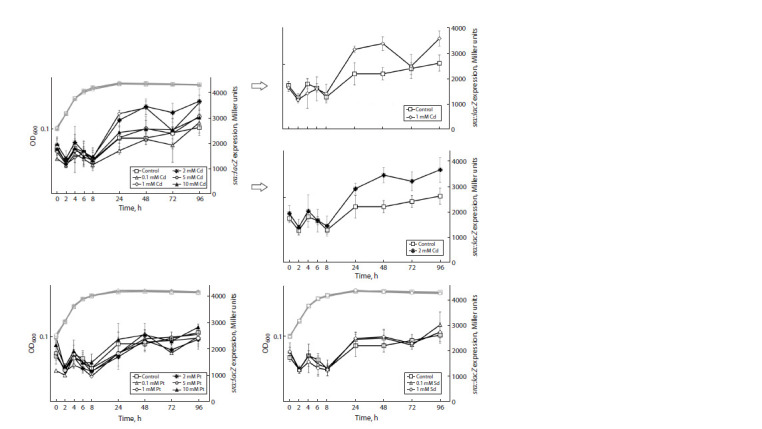
The influence of polyamine additions – putrescine (Pt), cadaverine (Cd) and spermidine (Sd) – at different concentrations on the expression of the sra gene at the translational level. OD600 – optical density at 600 nm.

According to the obtained data, the expression of the sra gene is also at a constantly high level at the stationary phase (Fig. 4). The expression of sra is noticeably increased by the addition of cadaverine at 1 mM and 2 mM. In this case, the maximal effect is observed at the stationary phase (48 h of cultivation). Additions of putrescine and spermidine have no effect on the sra expression.

**Fig. 5.**
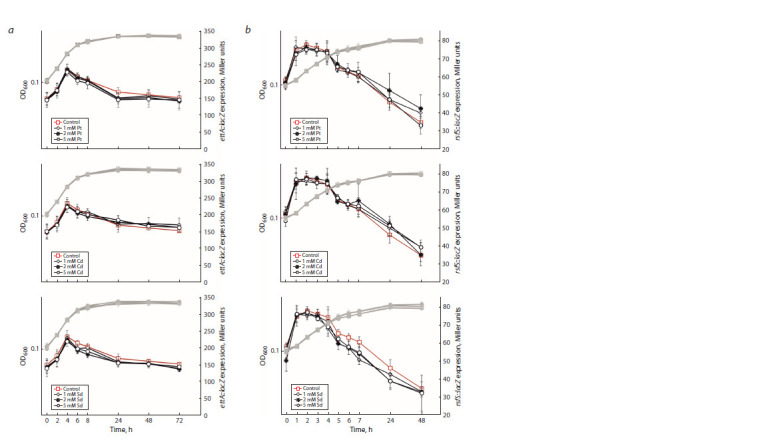
The influence of polyamine additions – putrescine (Pt), cadaverine (Cd) and spermidine (Sd) – at different concentrations on the expression of the ettA (a) and rsfS (b) genes at the translational level. OD600 – optical density at 600 nm.

In contrast to the above-mentioned genes, the maximal expression of the ettA and rsfS genes is observed at the exponential phase (Fig. 5). The highest expression for ettA occurs at 4 h of cultivation, and for rsfS, at 1–3 h. Polyamine supplements have no effect on the expression of these genes.

**Fig. 6.**
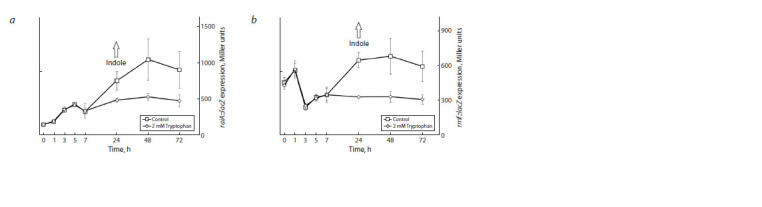
The influence of indole on the expression of the raiA (a) and rmf (b) genes at the translational level.

We have previously shown that the addition of 2 mM tryptophan at 0 h of cultivation is equivalently converted into indole at 24 h. In this case, the indole content during 7 h was detected to be at a low level, similar to the control, and increased dramatically at 24 h (Khaova et al., 2022), because the gene of the tryptophanase TnaA, catalyzing the formation of indole from tryptophan, is expressed in a RpoS-dependent manner (Li, Young, 2013; Gaimster et al., 2014). Under these conditions, we have previously studied the expression of a number of genes responsible for adaptation of stress in E. coli at the transcriptional level. The expression of only two genes, raiA and rmf, was elevated in response to an increase in indole content (Khaova et al., 2022). In this regard, we investigated the effect of indole on the expression of these genes at the translational level under the same conditions (Fig. 6). The results demonstrate that despite the stimulatory effect at the transcriptional level, the expression of both of these genes at the translational level dropped with the increase in the indole content, starting from 24 h of observation.

## Discussion

The functions of ribosome hibernation factors are to reversibly inhibit such a resource intensive process as translation under starvation and other stresses. These factors are known be under the control of master regulators (p)ppGpp, RpoS, CRP-cAMP responsible for the adaption of bacteria to multiple stressors at the stationary phase. Due to this, ribosome hibernation factors predominantly function at this period. However, during exponential growth, these factors are also able to maintain a base level of inactive ribosomes (Prossliner et al., 2018). The results we obtained show that most expression of the rmf, raiA and sra genes is observed exactly at the stationary phase (Table 3). In contrast, the ettA and rsfS genes demonstrate the maximal expression during exponential growth. The obtained results show that polyamines affect the expression of the “stationary-phase genes” rmf, raiA and sra, but not ettA and rsfS. In all cases, the stimulatory effect of polyamines is observed at the stationary phase. Thus, polyamines are able to induce the expression of genes responsible for the adaptation to stress, and thereby contribute to the formation of an adaptive state of a bacterial cell for the stationary phase. Moreover, the stimulatory effect is specific for the type of polyamine. The expression of each gene we studied depends on certain polyamines. The gene expression was predominantly positively modulated by putrescine and cadaverine.

**Table 3.**
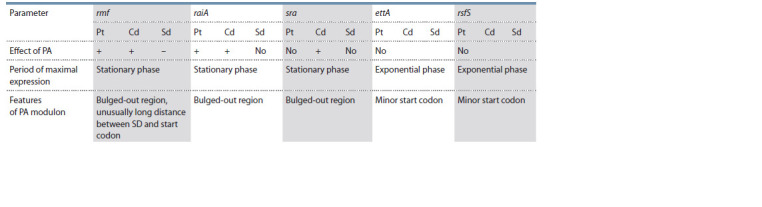
Effects of polyamines on the expression of the rmf, raiA, sra, ettA, rsfS genes at the translational level Notе. “+” – stimulating effect, “–” – inhibitory effect, “no” – no effect. PA – polyamine, Pt – putrescine, Cd – cadaverine, Sd – spermidine, SD – Shine–Dalgarno sequence

Although indole stimulated the expression of the rmf and raiA genes at the transcriptional level (Khaova et al., 2022), the results obtained in this work show its significant inhibitory effect at the translational level under the same conditions. This may indicate the possibility of a post-transcriptional mechanism for regulation of gene expression.

## Conclusion

The analysis of the mRNA primary structure of the rmf, raiA, sra, ettA, rsfS genes, encoding ribosome hibernation factors, as well as the obtained models of mRNA secondary structures showed that the studied genes have features of the polyamine modulon. We constructed strains harboring the translational lacZ-fusions and studied gene expression upon addition of the polyamines putrescine, cadaverine, and spermidine at different concentrations. These genes, with the exception of rmf, were studied for the effect of polyamines on their expression for the first time. The stimulatory effect of polyamines was observed at the stationary phase and was specific to the type of polyamine. Polyamines affected the expression of the rmf, raiA and sra genes, active at the stationary phase, but not ettA and rsfS, in which the highest expression was observed during exponential growth. Moreover, it was found that indole in-hibits the expression of the raiA and rmf genes at the translational level, despite positive modulation at the transcriptional level, which may indicate the possibility of a post-transcriptional regulation of the gene expression. The results obtained open up prospects for further research in this direction.

## Conflict of interest

The authors declare no conflict of interest.
